# Normal right- and left ventricular volumes and myocardial mass in children measured by steady state free precession cardiovascular magnetic resonance

**DOI:** 10.1186/1532-429X-11-19

**Published:** 2009-06-21

**Authors:** Emanuela Valsangiacomo Buechel, Thomas Kaiser, Clare Jackson, Achim Schmitz, Christian J Kellenberger

**Affiliations:** 1 Division of Pediatric Cardiology, University Children's Hospital, Zurich, Switzerland; 2 Division of Anesthesiology, University Children's Hospital, Zurich, Switzerland; 3 Departement of Diagnostic Imaging, University Children's Hospital, Zurich, Switzerland

## Abstract

**Background:**

Quantification of ventricular volume by Steady State Free Precession (SSFP) cardiovascular magnetic resonance is accurate and reproducible. Normal values exist for adults, but are lacking for children.

We sought to establish normal values for left and right ventricular volumes, mass and function in healthy children by using SSFP.

**Methods and results:**

Fifty children (27 females, 23 males) without cardiovascular disease were evaluated. Median age was 11 years (range 7 months – 18 years), weight 35 kg (range 7–77 kg), height 146 cm (range 66–181 cm). Thirty-six examinations were performed with breath holding, 14 in freely breathing sedated children.

Ventricular volumes and mass were measured in the end systolic and end diastolic phase on SSFP cine images acquired in a short axis plane as a stack of 12 contiguous slices covering full length of both ventricles.

Regression analysis showed an exponential relationship between body surface area (BSA) and ventricular volumes and mass (normal value = a*BSA^b^). Normative curves for males and females are presented in relation to BSA for the enddiastolic volume, endsystolic volume and mass of both ventricles. Intra- and interobserver variability of the measurements was within the limits of 2% and 7% respectively, except for right ventricular mass (10%).

**Conclusion:**

The exponential equation for calculation of normal values for each ventricular parameter and graphical display of normative curves for data acquired in healthy children by SSFP cardiovascular magnetic resonance are provided.

## Background

Cardiovascular magnetic resonance (CMR) is nowadays considered an essential tool for advanced assessment of congenital heart disease (CHD). Besides anatomical information, CMR can provide reliable quantification of various functional cardiac parameters. CMR measurements of ventricular volumes, mass and function have been validated in vitro and in vivo, demonstrating excellent accuracy and reproducibility of the method [1,2]. Thus CMR is considered superior to any other technique and often cited as the "gold-standard" [3]; its results are getting increasingly used for clinical decision making [4]. This is especially true for the right ventricle (RV) and an abnormally shaped left ventricle (LV), which are of particular interest in CHD, as the measurement used in CMR is not based on geometrical assumptions, but reproduces the real shape of a certain volume (disc summation method) [5].

The Steady State Free Precession (SSFP) cine sequence is the technique used in most centres for single-slice multi-phase acquisitions [6,7]. The superior discrimination between blood and endocardium by using SSFP results in different volume measurements compared to gradient echo cine images; therefore different normal volumetric values are required [8].

Normal values for the left and the right ventricle in the adult population have been published [9,10], but normal data for children are scarce, and have mainly been acquired with the previously used gradient echo cine sequence in only a small number of subjects [11,12]. In paediatric patients, CMR data regarding the ventricular volumes are increasingly used for critical clinical decision making, such as indication for uni- or biventricular repair in complex CHD or for deciding on reinterventions during follow up of repaired CHD. Therefore, establishment of volumetric normative data for children with the currently used CMR technique is urgently needed.

The aim of our study was to establish normal values for left and right ventricular volumes, mass and function in healthy children by using CMR with SSFP sequences.

## Methods

### Subjects

Fifty-two subjects were recruited for the study among physically normally developed children without cardiovascular disease, undergoing magnetic resonance imaging for evaluation of peripheral and local anomalies, typically orthopaedic disease. Inclusion criteria were age between 0 and 18 months and no evidence of cardiovascular disease. Exclusion criteria consisted of presence of disease affecting the chest or the cardiovascular system, acute infections, arterial hypertension, arrhythmias, anaemia, neoplasm, and use of any drug. An abnormal body size for the age, defined as bodyweight or length below the 3^rd ^or above the 97^th ^percentile of the normative values for the Swiss population, represented an additional exclusion criterion [13]. Two children were excluded for abnormal body size according to this criterion.

Written informed consent for participation in the study was obtained from the parents in all cases and from the child whenever possible. The patients demographic data as well as bodyweight and height where collected at the same day of the examination.

The ethics board of our institution approved the study protocol.

### Technique

The magnetic resonance examination was performed on a 1.5 Tesla scanner (Signa HDx, GE Medical Systems, Milwaukee, WI, USA). CMR was performed after completion of the clinical MR examination by using an 8-channel phased-array cardiac coil for larger children and a quadrature head coil for smaller children. Cine imaging was performed with a two dimensional retrospectively cardiac gated SSFP sequence (2D FIESTA) using following parameters: TR 3.5–4.2 ms, TE 1.5–1.8 ms, flip-angle 45°, bandwidth ± 125 kHz, matrix 224 × 224, number of excitations 1 (breath hold) or 2 (free breathing), field of view 250–350 mm, field of view in phase direction 75%, slice thickness 5–8 mm with a gap of 0–2 mm depending on body size and targeting 12 slices to be acquired. If Gadolinium based contrast medium had been previously given for the clinical study, the flip angle was raised to 60° in order to optimise the contrast between blood and myocardium.

The degree of k-space segmentation (views per segment 4–12) and number of images reconstructed per cardiac cycle (20 – 30) was adjusted to the heart rate in order to achieve a true temporal (calculated as TR × views per segment) resolution in the range of 21–45 ms, for accurate definition of the end-systolic and end-diastolic phase[14,15]. Thus mean temporal resolution obtained was 34 ± 7 ms.

After obtaining sagittal, coronal and axial localizers, SSFP cine images were acquired first in the vertical and horizontal long axis planes for planning the subsequent stack of at least 12 contiguous slices in the short axis plane covering both ventricles in their entire length. The short axis images were obtained from the base to the apex of the heart, with the most basal slice positioned parallel to and across the plane of the atrioventricular valves [10,16].

Non-sedated children were instructed on how to hold their breath prior to the examination, and the images were acquired during end-expiratory breath holds. In children examined under sedation the images were acquired during free breathing.

### Image analysis

All images were analysed on a commercially available off-line workstation (SUN Microsystems Inc., Mountainview, CA, USA). Measurement of ventricular volumes and myocardial mass was performed using the MASS+ Software package (Magnetic Resonance Analytical Software System, Version 4.0, MEDIS, Medical Imaging Systems, Leiden, The Netherlands).

The end-systolic and the end-diastolic phase were first identified visually on a movie loop of a midventricular slice. The endocardial contours were then traced manually on the images of the estimated end-systolic and end-diastolic phase, as well as on the two previous and following phases, for a correct detection of the minimal (end-systolic) and maximal (end-diastolic) volume. Stroke volume was calculated as the difference between the end-diastolic volume (EDV) and the end-systolic volume (ESV) and ejection fraction (EF) was defined as EDV-ESV/EDV.

**Figure 1:**
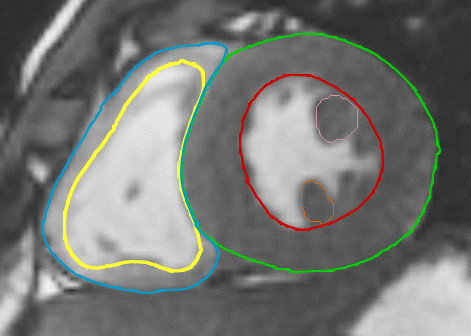
**Contour tracing in the endsystolic phase**.

The epicardial contours were traced in the end-systolic and in the end-diastolic phase for calculation of the ventricular mass. The myocardial volume was calculated as the difference of the epicardial and the endocardial volumes and multiplied by the specific density of the myocardium (1.05 g/ml) for obtaining the value of myocardial mass[17]. For calculation of global ventricular volumes, mass and function the papillary muscles were included in the ventricular cavity (figure 1). Similarly, the myocardial trabeculations of the right ventricle [18] and the moderator band were included in the RV cavity. Finally, the mass of the papillary muscles of the LV was analysed separately and presented in a different normogram (figure 3).

For correct identification of the most basal slice, i.e. if the slice was located in the atrium or in the ventricle, cavities surrounded by at least 50% of their circumference by myocardium were considered as ventricular [19]. In addition the observation of the changes in volume (dilatation or contraction) during systole or diastole of a cardiac structure helped to identify the structure as atrium or ventricle. Ventricular volume was calculated by summation of the traced ventricular cavity areas, multiplied by the sum of slice and gap thickness.

### Sedation Procedure

Anaesthesia was induced either intravenously with propofol 2 mg/kg and further doses of 1 mg/kg as necessary to tolerate positioning, or by inhalation with sevoflurane 4–8% in oxygen/nitrous oxide (1:1) until an intravenous line was established (Aestiva 5/MRI, Datex-Ohmeda, Helsinki, Finland). Meticulous care was taken to maintain spontaneous breathing. 2 L/min Oxygen was applied with a nasal cannula with separate O_2 _tube and CO_2 _sampling tube (Salter Labs, Arvin, California). Deep sedation was continued with propofol 10 mg/kg/h given as continuous intravenous infusion, following our institutional protocol. Routine monitoring of capnography with respiratory rate and end-tidal carbon dioxide concentration, oxygen saturation, heart rate, electrocardiogram and blood pressure, was assessed continuously (Datex-Ohmeda MRI Monitor, Datex-Ohmeda, Helsinki, Finland).

### Statistical analysis

Demographic data are expressed as median and range. Body surface area was calculated from the Mosteller formula[20].

Several regression models were used to examine the allometric relationship between ventricular parameters and BSA. We tested the following equations: Y = ax+c, Y = ax^b^, Y = ax^2^+bx+c and Y = ax^3^+bx^2^+dx+c, where Y is the predicted value and x is BSA. For exponential functions, a logarithmic transformation of the measurements was performed before fitting of the regression models for the purpose of stabilize variances. The best fitting model was defined by a large R^2^, i.e by a high percentage of the variability of the data that is explained by the association between variables, and analysis of residuals for each regression equation. The resulting equations and parameters were then retransformed to a linear scale. Significance of gender, previous administration of contrast-medium, and acquisition of the images during free-breathing or breath holding were tested by introducing a factor accounting for each of these variances in the regression model. A p < 0.05 was considered statistically significant. Intra- and interobserver variability were evaluated in two observers blinded to each other by using Bland-Altman analysis in 10 randomly selected patients[21] Statistical analysis was performed using commercially available software packages (Prism 4.03, GraphPad Software Inc. San Diego USA and SPSS for Windows, Rel. 14.0.1. 2005. Chicago: SPSS Inc.).

## Results

### Demographics

50 normotensive children (23 male, 27 female) were included in the study. Demographic data are shown in table 1. No significant difference was found for body size between males and females.

**Table 1:** Demographic data expressed as median (range).

	All (n = 50)	Males (n = 23)	Females (n = 27)	
Age (years)	11 (0.7 – 18)	11 (0.7 – 16)	10 (0.7 – 18)	n.s
Weight (kg)	35 (7–69)	36 (7 – 69)	33 (8 – 60)	n.s
Height (cm)	146 (66 – 181)	147 (67 – 181)	144 (66 – 170)	n.s
BSA (m2)	1.18 (0.37 – 1.85)	1.21 (0.37 – 1.85)	1.18 (0.38 – 1.67)	n.s
Heart rate	84 (55–127)	78 (55–99)	88 (58–127)	n.s

In 36 children image acquisition was performed during breath holding; 13 were examined in deep sedation and images acquired during free breathing; one child was unable to hold his breath and images were acquired in free breathing as well.

### Volumetric parameters

The regression model y = ax^b ^best described the relationship between ventricular parameters and BSA. Thus the final normative equation for all ventricular parameters was: normal value = a*BSA^b^.

All values for each ventricular parameter are described in table 2. Despite the similar body size and BSA between both genders, significantly larger systolic and diastolic ventricular volumes were found in males; in contrast, no gender difference was observed for cardiac output and ejection fraction (table 2). In contrast, previous administration of gadolinium and image acquisition during breath holding or during free breathing did not show any statistical significance; therefore the corresponding factors could be eliminated from the regression model and do not figure in the final equation. Measurements of myocardial mass in endsystolic and endiastolic phase did not differ significantly, and only data from enddiastolic phase are shown. On the base of normal data, z-values can be calculated for each performed measurement by using the standard deviation (SD) given in table 2 and following equation adapted for the logarithmic transformation (described in the methods section):

**Table 2:** Parameters for gender-specific equation (normal value = a *BSA^b^).

	a males	a females	b	SD	r^2^	p-value
LVEDV (ml)	77.5	67.8	1.380	0.0426	0.98	<0.00005
LVESV (ml)	29.7	26.1	1.370	0.0647	0.95	<0.005
LVSV (ml)	47.4	41.7	1.394	0.0500	0.97	<0.0005
LVCO (ml/min)	3890	3622	1.062	0.0727	0.89	ns
LVM (g)	53	45.2	1.304	0.0475	0.97	<0.00005
Papillary muscle (g)	1.9	1.6	1.451	0.0976	0.85	<0.05
RVEDV (ml)	83.8	72.7	1.469	0.0499	0.97	<0.0001
RVESV (ml)	35.3	30.2	1.559	0.0737	0.95	<0.005
RVSV (ml)	48.2	42.1	1.407	0.0524	0.97	<0.0005
RVCO (ml/min)	3947.3	3658.3	1.076	0.0783	0.88	ns
RVM (g)	16.7	14.9	1.331	0.0605	0.95	<0.01

For practical reasons, the gender factor has been integrated in the a value, yielding to different a values for males and females. LVEDV: left ventricular enddiastolic volume; LVESV: left ventricular endsystolic volume; LVSV: left ventricular stroke volume; LVCO: left ventricular cardiac output; LVM: left ventricular myocardial mass; RVEDV: right ventricular enddiastolic volume; RVESV: right ventricular endsystolic volume; RVSV: right ventricular stroke volume; RVCO: right ventricular cardiac output; RVM: right ventricular myocardial mass.



**Figure 2:**
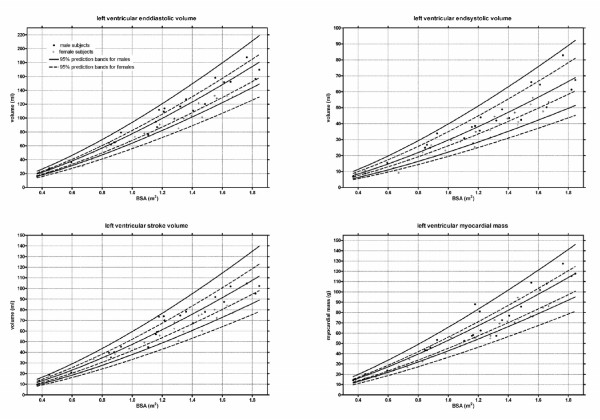
**Left ventricular volumes and mass**. Lines indicate median and 95% prediction bands.

**Figure 3:**
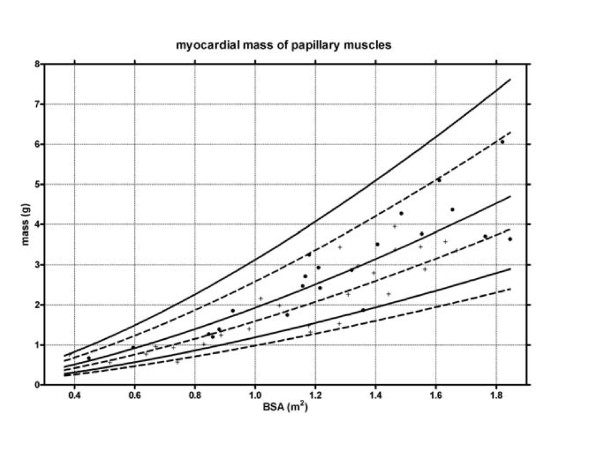
**Mass of the papillary muscles**. Lines indicate median and 95% prediction bands.

**Figure 4:**
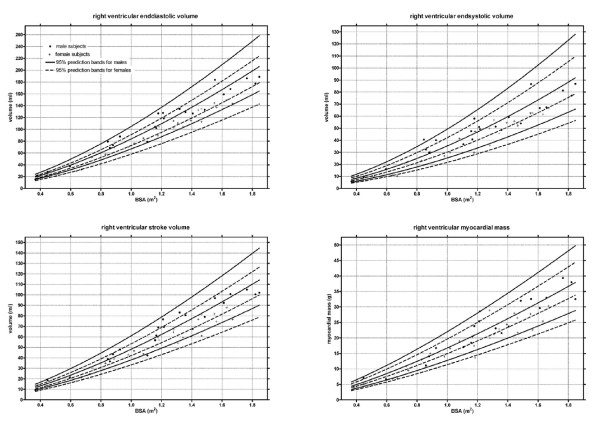
**Right ventricular volumes and mass**. Lines indicate median and 95% prediction bands.

Figure 2 and 3 shows the gender specific normative curves for the ventricular volumes and mass of the left ventricle, and figure 4 for the right ventricle. The EF of the left ventricle was constant for all BSA, with a mean EF of 61.3% (SD 4.1%). In contrast, a slightly negative correlation was found between BSA and the right ventricular EF, fitting following regression equation EF = 61.8 - 3.6 * BSA (SD 4.2).

### Reproducibility

Intra- and interobserver variability is summarized in Table 3. Variability was slightly smaller for measurements of the left ventricle with a variability range from 1% to 3% for intraobserver measurements, and from 2% to 7% for interobserver measurements. Variability for measurements of the right ventricle ranged from 2.6% to 9% for intraobserver assessment, and from 4% to 10% for interobserver assessment.

**Table 3:** Inter- and intraobserver variability.

	**Interobsverser variability**			**Intraobserver variability**		
	mean difference	limits of agreement	coefficient of variability %	mean difference	limits of agreement	coefficient of variability %

LVEDV (ml)	1.8	-1.7/5.2	2.3	-0.1	-2.3/2.0	1.4
LVESV (ml)	2.5	-1.8/6.8	7.1	0.0	-1.6/1.6	2.8
LVM (g)	-5.6	-11.4/0.3	6.3	-0.5	-3.2/2.2	2.8
LVEF (%)	-2.6	-7.9/2.6	4.5	-0.2	-1.7/1.3	1.3
RVEDV (ml)	0.7	-6.2/7.5	4.2	-2.3	-6.5/1.9	2.6
RVESV (ml)	0.2	-3.4/3.9	5.4	-0.5	-3.1/2.0	3.8
RVM (g)	-3.2	-5.8/-0.5	10.1	1.0	-2.0/3.9	9.4
RVEF (%)	0.0	-5.0/5.1	4.4	-0.5	-4.6/3.5	3.5

LVEDV: left ventricular enddiastolic volume; LVESV: left ventricular endsystolic volume; LVM: left ventricular myocardial mass; LVEF: left ventriculat ejection fraction; RVEDV: right ventricular enddiastolic volume; RVESV: right ventricular endsystolic volume; RVM: right ventricular myocardial mass; RVEF: right ventricular ejection fraction

## Discussion

This study provides normal values for the left and right ventricular volumes, myocardial mass, and ejection fraction in children and adolescents measured by using the SSFP sequence. These data are an update to previously published normative values which were collected only in small paediatric sample sizes and had been acquired with the gradient-echo cine sequence, which is not being commonly used any more [11,16]. Maceira et al have published recently normal values acquired with the SSFP technique for the adult population [9,10]. Their data were acquired in subjects older than 20 years of age and showed that in adults most ventricular parameters are significantly and independently influenced by gender, age and BSA [9,10]. We present paediatric reference values, covering an age spectrum from 1 to 18 years and taking into account the correct allometric relationship between cardiac dimensions and body size in the growing body [22]. Notably, in growing individuals the correlation between age and BSA is more pronounced than in adults.

Our data can be compared with those of a very recent publication. Robbers-Visser et colleagues established volumetric normative data in a similar number of healthy children [23]. They used the same acquisition technique and similar analysis algorithm for the images, but a different statistical method by correlating the data to BSA with a univariate linear regression analysis. Even by considering this methodical difference, the results of both studies fit together precisely, with an insignificant difference of 2 ml/m^2 ^for the left ventricular EDV and of 4 ml/m^2 ^for the right ventricular EDV. Additionally, following characteristics of the data has been found by both groups: gender modifies the relationship between volumetric data and BSA, so that gender needs to be added as separate variable in regression models; ejection fraction remains constant throughout growth and does not differ significantly between boys and girls; a good R^2 ^points out a strong relationship between BSA and ventricular volumes; SSFP images yield to larger volume measurements than gradient echo cine images, as previously demonstrated [8,24]. If we compare our results with those obtained previously with the gradient echo cine technique by the same group [11], we found slightly larger end-diastolic and end-systolic volumes for both ventricles (LVEDV +9 ml/m^2^, RVEDV +14 ml/m^2^), resulting in a difference in stroke volumes less than 1 ml.

Finding the correct allometric relationship is crucial for exact description of growth of the human heart in relation to BSA [22]. We used an exponential equation with exponents for BSA ranging from 1.3 (LV mass) to 1.56 (RVESV), which is in agreement with the existing literature. Sluysmans et al advocated, after extensive model analysis, the use of a two-parameter regression model (Y = ax^b^) with an exponent for BSA of 1.385 for calculation of LVEDV, measured by 2D echocardiography [22]. Lange et al in an angiographic study found exponents for BSA ranging from 1.17 (RVEDV) to 1.34 (LVESV)[25]. Finally Gutgesell et al in a meta-analysis of published echocardiographic and angiographic data described the appropriate regression equation Y = 70*5BSA^1.4 (1.3–1.5) ^for determination of LVEDV[26]. The high R^2 ^(≥ 0.95 for volumetric data) obtained in our study by using an exponential equation for BSA and introducing an additional factor for gender, demonstrate that the fitting model is appropriate and more accurate than others used in different studies [23].

On the base of all these data demonstrating that cardiac volumes have a non-linear relation to body surface area, and since the exponential values are different for different cardiac parameters, it would not be appropriate to provide normal values simply indexed to BSA in a table [26]. Therefore, we decided to provide the appropriate regression formula for every normal value that needs to be calculated. For practical and clinical use, we provide an additional graphical display of the normal curve for each parameter and gender in relation to BSA, which is the recommended and commonly used parameter for body size in paediatrics.

The correct definition of the endocardial contours has a major impact on the accuracy of volumetric measurements. It has been shown that delineation along the border of the compact myocardium has better reproducibility than delineation along the fine endocardial trabeculations [18]. Sievers et al found small systematic but significant differences on volumetric measurements, if the papillary muscles were delineated separately, which they judged to be of no clinical relevance [27]. We excluded the papillary muscles from the ventricular mass for the overall measurements, and provide separate data for the papillary muscles allowing calculation of full myocardial mass.

The intra- and inter-observer variability in our study are within the variability range previously described in the literature confirming the good reproducibility of the CMR volumetric measurements [11,23,28]. Similarly to others, we found slightly larger variability for measurements of the right ventricle, likely due to the difficult definition of the plane of the atrioventricular valve, the fine course trabeculations and partial volume effects inherent of the right ventricle [11,18]. For the same reasons, the highest variability was found for measurements of the right ventricular mass. In addition, being the right ventricular myocardium very thin, its proper delineation may be challenging in children. Therefore, quantification of right ventricular mass in children should be performed with caution and investigators need to be aware of its limited reproducibility.

### Limitations

Small children recruited for the study (n = 14) were examined under sedation with propofol and the images were acquired while freely breathing. Due to respiratory motion, the image quality was not as good as when acquired during breath holding. Nevertheless, the cardiac borders were perceived well enough for allowing tracing of the contours in all children. The image quality and accuracy of volume measurements can be improved in small children by performing the CMR exam under general anaesthesia with controlled ventilation allowing image acquisition in breath hold. Due to obvious ethical reasons, we could not intubate any young child solely for investigational purposes. When performing studies in sedation or general anaesthesia, investigators need to be aware that anaesthetic drugs may have some minor influence on cardiac function

In our study group we could not find any statistical significant difference between the regression curves of subjects studied during free breathing (sedation) and those who were able to held their breath; therefore the two groups are presented in the same normative curve.

More detailed analysis of the effects of gender, BSA, weight, height, and conditions of image acquisition, such as breath hold or free breathing, sedation or mechanical ventilation, on the ventricular volumetric values, requires a larger number of subjects studied. In the field of paediatric cardiology, these results may be achieved by planning a large multi-centre study.

## Conclusion

This study provides the normal values for left- and right ventricular volumes, mass and function in children, determined by SSFP CMR. As the relationship between cardiac volumetric data and BSA is not linear, the data are displayed graphically in form of normative curves and the appropriate equations for calculation of the expected value are provided for each gender and parameter. These data are of significant clinical and research utility for centres assessing children with congenital or acquired heart disease by CMR.

## Competing interests

The authors declare that they have no competing interests.

## Authors' contributions

EVB conceived and designed the study and gave a major contribution to the draft of the manuscript and its revision. TK carried out subject's recruitment, image acquisition and analysis and drafted the manuscript. CJ was involved in data analysis and advanced statistical calculation. AS was responsible for children's sedation, helped in coordinating the study and contributed to the manuscript draft. CJK helped in designing the study, image acquisition and participated in the revision of the manuscript.

All authors read and approved the final manuscript.
